# New User-Friendly Approach to Obtain an Eisenberg Plot and Its Use as a Practical Tool in Protein Sequence Analysis

**DOI:** 10.3390/ijms12095577

**Published:** 2011-08-30

**Authors:** Rob C.A. Keller

**Affiliations:** Section Chemistry, Charlemagne College, Wilhelminastraat 13-15, 6524 AJ Nijmegen, The Netherlands; E-Mail: rcakeller@kpnmail.nl; Tel.: +0031-243820460; Fax: +0031-243820461

**Keywords:** amphitropic proteins, Eisenberg plot, hydrophobic moment plot, Heliquest, lipid binding regions, protein-lipid interactions, transmembrane proteins

## Abstract

The Eisenberg plot or hydrophobic moment plot methodology is one of the most frequently used methods of bioinformatics. Bioinformatics is more and more recognized as a helpful tool in Life Sciences in general, and recent developments in approaches recognizing lipid binding regions in proteins are promising in this respect. In this study a bioinformatics approach specialized in identifying lipid binding helical regions in proteins was used to obtain an Eisenberg plot. The validity of the Heliquest generated hydrophobic moment plot was checked and exemplified. This study indicates that the Eisenberg plot methodology can be transferred to another hydrophobicity scale and renders a user-friendly approach which can be utilized in routine checks in protein–lipid interaction and in protein and peptide lipid binding characterization studies. A combined approach seems to be advantageous and results in a powerful tool in the search of helical lipid-binding regions in proteins and peptides. The strength and limitations of the Eisenberg plot approach itself are discussed as well. The presented approach not only leads to a better understanding of the nature of the protein–lipid interactions but also provides a user-friendly tool for the search of lipid-binding regions in proteins and peptides.

## 1. Introduction

The Eisenberg plot or hydrophobic moment plot is one of the most beautiful examples of where bioinformatics really started off. In the search for methods to translate the primary sequence into more advanced structural information about the structure and folding of proteins, Eisenberg and co-workers developed their methodology [1,2]. Over the past two-three decades, it has become one of the most frequently used approaches in bioinformatics. In essence, the Eisenberg plot pictures the mean hydrophobicity (a measure for the overall hydrophobicity of the sequence) against the mean hydrophobic moment (a measure for the way polar and non-polar amino acids in the sequence are distributed). With the use of the so-called normalized consensus scale, both parameters of a sequence are calculated and windows of varying length between 7–20 amino acids are reported in the literature [2]. The way in which hydrophobicity is fluctuating along a sequence within a protein can be calculated and plotted also in a modified approach [3]. Whether a protein sequence region belongs to a globular, surface seeking or transmembrane protein is a frequently used application of the Eisenberg plot methodology (see [4] for a review). Particulary the search for surface seeking regions in proteins and peptides has received a lot of attention [5]. More recently approaches have been developed that have a special feature to recognize lipid binding regions in proteins [6–8].

Lipids and lipid–protein interactions play an increasingly appreciated and recognized role in many biological processes (see for reviews [9–11]). One interesting recent development is the bioinformatics approach, which enables the identification of lipid binding helical regions in proteins using the Heliquest web-server [6]. A recent example of this approach has been demonstrated for protein translocation motor proteins [12] with the identification of a possible general feature of these motor proteins: the possession of multiple lipid binding regions. The recent finding that multiple lipid binding regions can be identified in a protein translocation motor protein like *E. coli* SecA [12] corresponds with and possibly expands the earlier findings that specific SecA-lipid interactions could be demonstrated using different approaches [13–15].

This briefly exemplifies the potential power of the Heliquest-based bioinformatics method [6,12]. A closer look at the Heliquest software suggests additional possibilities of this program for the use in the Eisenberg plot methodology since the Heliquest software gives details about, the net charge (*z*), the mean hydrophobicity (<*H*>) and the mean hydrophobic moment (μ*H*). In this study the Heliquest approach, though specialized in identifying lipid binding helical regions in proteins, was used to obtain the “original” Eisenberg plot. For this purpose the influence of using another hydrophobicity scale, the Fauchere and Pliska scale [16] instead of the normalized scale of Eisenberg [2], was examined. This study indicates that the Eisenberg plot methodology can be transferred to another hydrophobicity scale and can provide a user-friendly approach. The relevance of this particular methodology is checked on a number of individual cases. The strength and limitations of the Eisenberg plot approach, alone or in combination with the Heliquest lipid-binding feature, are discussed as well.

## 2. Results and Discussion

### 2.1. The Eisenberg Plot Approach Using the Original Databases

The Eisenberg plot methodology used an algorithm for detecting hydrophobic polypeptide sequence segments and discriminates between surface-seeking and transmembrane regions. This study checked whether the Heliquest data can give valid results according to the Eisenberg plot methodology [1,2], and whether various regions in a polypeptide could be divided by boundary lines, resulting in three possible alpha-helical properties: transmembrane, lipid surface-seeking and globular. In order to detect whether the data obtained by the Heliquest program allow detection of possible lipid membrane binding and hydrophobic motifs according to the Eisenberg plot methodology, the original databases were investigated [1,2]. For this purpose the corresponding sequences were run through the Heliquest program.

**Figure 1:**
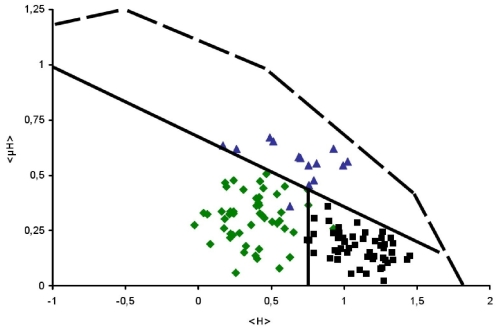
Eisenberg plot as obtained by Heliquest generated data based on the original databases of Eisenberg and co-workers [1,2]. The originally identified Globular ( 
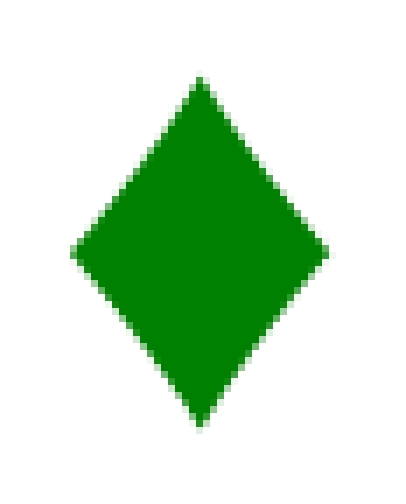
), Surface seeking ( 
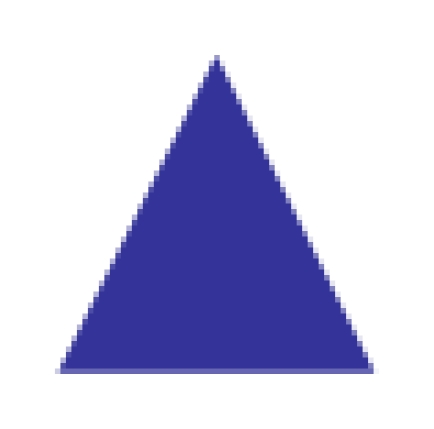
) and TransMembrane (■) segments are depicted.

The results found with Heliquest generated data (Figure 1) correspond well with the overall picture of the original Eisenberg approach (see Table S1 and Table S2 for detailed description of all data used). This indicates that the data obtained by the Heliquest program are applicable and that the use of another hydrophobicity scale [16] with the Heliquest generated Eisenberg plot approach is valid. Obviously the scale and absolute numbers for the individual segments differ due to the use of this other hydrophobicity scale. It is interesting to note that the surface seeking regions can be distinguished even better by the Heliquest generated approach than in the original plots.

In the original Eisenberg plot methodology two features were extracted. First of all, a surface seeking propensity for surface helices are thought to exist for points close to the line <μ*H*> = 0.600 – 0.342 <*H*>. Secondly, potential transmembrane helices are assumed if the mean hydrophobicity *<H*> is greater than 0.51 and the mean hydrophobic moment is below the line as defined above [2]. The corresponding features in the plot obtained by Heliquest generated data are <μ*H*> = 0.654 – 0.324<*H*> and *<H*> above 0.75 respectively.

### 2.2. The Validity Check of the “New” Eisenberg Plot

In order to check the validity of the newly obtained Eisenberg plot one step further, a number of more recent examples were checked which were not included in the Eisenberg databases [1,2]. In Table 1, a number of examples are depicted with more recent data that used the original Eisenberg approach and which were compared with the Heliquest generated Eisenberg plot.

**Figure 2:**
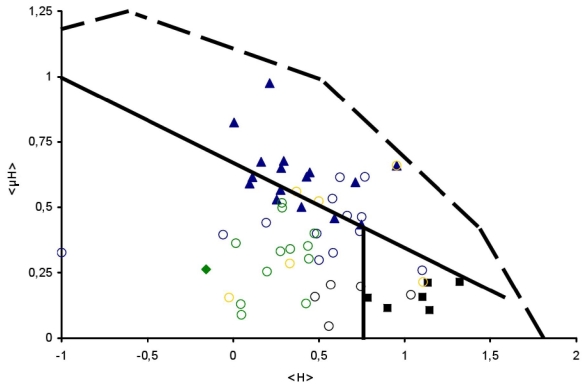
Eisenberg plot of a number of successfully identified proteins and peptides in which Surface seeking ( 
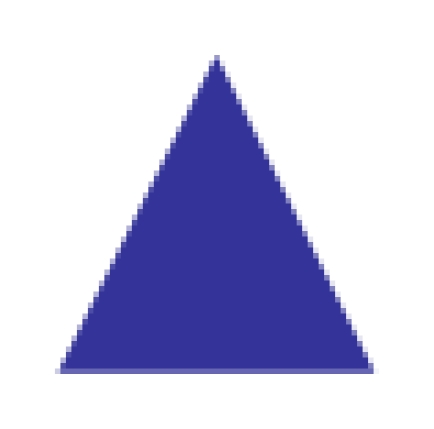
), Globular ( 
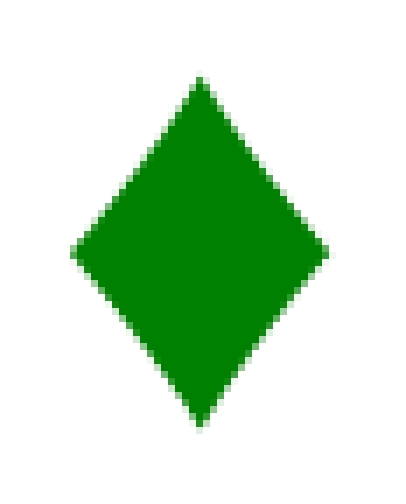
) and TransMembrane (■) segments are depicted, see Table 1 for details. Examples of signal peptides (SP) (circles, black), lipid-binding peptides (LBP) (circles, blue), amphitropics (circles, green) and others (circles, orange) are depicted, see Table 2 for details.

All data confirmed the findings obtained with the original Eisenberg approach (see Figure 2), which strongly substantiates the applicability of the Heliquest generated hydrophobic moment plot methodology. For example all surface seeking (S) regions of proteins and peptides were identified as such in the Heliquest generated approach and are found situated in or close to the surface seeking area of the Eisenberg plot.

It has previously been discussed that the Heliquest lipid binding discrimination factor, when used in the analysis mode, cannot be used to identify transmembrane regions [12]. According to the results depicted in Table 1, it is clear that the Eisenberg methodology identified the transmembrane regions, since the Heliquest generated <*H*> is in all these cases above 0.75. Additionally the lipid discrimination factor D identified a substantial amount of all depicted (Table 1) experimentally demonstrated lipid binding regions [17–29]. The combination of the Heliquest discrimination factor and the Heliquest generated Eisenberg plot data was able to predict and identify all potential lipid binding regions. For example the lipid binding capability of WALP23 [26] is missed by the Heliquest discrimination factor but is recognized as transmembrane region by the Heliquest generated Eisenberg plot approach. The lipid binding capacity of Histatin 5 [21] is not identified by the Eisenberg plot approach but is well recognized by the Heliquest discrimination factor. This strongly suggests that in general the confirmative value of the combination of these two approaches would be even higher than the already impressive positive prediction value of 86% of the Heliquest discrimination factor alone [6].

### 2.3. The Meaning of the Eisenberg Plot for Novel Classes of Proteins and Peptides

The results obtained using the Heliquest generated Eisenberg plot methodology demonstrated it to be a valid and equally powerful approach as compared to the original Eisenberg plot methodology. However, over the last two decades numerous examples of experimentally demonstrated lipid-binding of proteins and peptides have been reported where the Eisenberg plot approach did not always identify them as either surface seeking or transmembrane [5,30].

In other words, there is evidence for novel classes or subclasses of proteins and peptides which cannot be classified as Globular, Surface seeking or Membrane protein. The data as depicted in Figure 2 and Table 2 used solely examples of experimentally demonstrated lipid binding of proteins and peptides [14,31–59]. For example some of the depicted signal sequences, all well described in literature for their ability to bind to (anionic) phospholipids [31–35], were found to be located in the globular protein region. The results of the depicted signal sequences obtained by the Heliquest generated data were found to correspond with the results as described and discussed in a thorough signal sequence analysis performed with the original Eisenberg plot methodology [60]. Thanks to the pioneer work of Von Heijne and co-workers, who performed statistical analysis of signal sequence and presequences [61–63], it is well-known that for example mitochondrial targeting sequences form amphiphilic helices and are identified by the Eisenberg plot methodology as surface seeking [61]. Eukaryotic signal sequences frequently can be found in the transmembrane region in an Eisenberg plot, probably due to their longer hydrophobic region compared to the signal sequences present in prokaryotic organisms [60,62,63]. Since the introduction of the hydrophobic moment plot methodology, numerous other novel peptides summarized as lipid binding peptides (LBP peptides) have been analyzed systematically by the Eisenberg approach. A few typical examples are depicted in Table 2, for example Aurein [36,37] a typical α-AMP peptide and penetratin [43] a typical cell penetratin peptide. A large number of these peptides were found to be located in the globular protein area of an Eisenberg plot. In the case of the α-AMP peptides, a specific area has been identified in the globular protein area of an Eisenberg plot where such peptides are often found and a possible use of this dedicated area for identification purposes has been postulated [5,39,64]. All sequences, being part of the amphitropic protein family, were not recognized by the Eisenberg plot methodology as either surface seeking or membrane protein, while the Heliquest lipid binding discrimination factor interestingly enough identified all these regions as lipid-binding. For protein translocation motor proteins multiple lipid-binding regions were predicted which are apparently required for a reversible membrane binding and proper functioning [12]. Multiple lipid binding were found in other amphitropic proteins like FtsY [12,49,50], and apocytochrome c [12,51] as well, indicating a specific feature of these members of the amphitropic protein family. It can be concluded that more recently recognized types of proteins and peptides that are classified as for example amphitropic, signal sequences or (α-) AMP peptide, cannot always be detected by the Eisenberg approach due to its novel and more complex features. Intriguingly, the Heliquest discrimination factor often identified the lipid binding regions in such proteins and peptides.

### 2.4. Examples Illustrating the Power of the Total Approach

This study indicated the power of the combined use of the Heliquest lipid binding discrimination factor and the Heliquest generated Eisenberg plot methodology. This aspect of the development of the most complete approach in the search for potential lipid binding regions was investigated for some additional examples.

The first example is the well-known and thoroughly studied M13 coat protein [65,66]. The Heliquest lipid binding discrimination factor identified clearly two predicted lipid-binding regions (Table 3). Additionally the Heliquest generated Eisenberg plot approach identified one of these regions as transmembrane. Both these predicted findings correspond well with what was demonstrated experimentally [65,66].

Since it has been demonstrated experimentally that FtsY contains lipid-binding regions [49,50], and recently novel lipid binding regions have been predicted [12], the closely related protein Ffh was investigated. There are no reports indicating the possible lipid-binding regions in Ffh, however there is some experimental evidence for an existing protein-lipid interaction when it comes to Ffh membrane binding (see [68,69]). The Heliquest discrimination factor identified multiple novel lipid binding regions in Ffh (Table 3), seeming divided over four lipid binding domains (LBD), regions ranging from AA 1–61, AA 166–183, AA 309–353 and AA 395–445. The Heliquest generated Eisenberg plot identified two possible binding regions as surface seeking helices, the lipid binding regions AA1–18 and AA415–432.

A recent report indicated that the cytosolic domain of Fis1 binds reversibly to lipids and might be another member of the rapid growing family of amphitropic proteins [67]. The Heliquest lipid binding discrimination factor identified four lipid binding regions (Table 3). The Heliquest generated Eisenberg plot analysis identified one possible lipid-binding region as surface seeking, region AA 35–52, and one lipid binding region as transmembrane, AA 133–150. Indeed the region AA 133–150 has been identified before as transmembrane [70] and upon binding to lipids a recent report about the cytosolic domain of Fis1 indicated a more non-polar environment for two Trp-residues, close to the AA 35–52 region.

## 3. Method Section

### 3.1. Primary and Secondary Structures Identification

The primary structure of the proteins was obtained from either the Swiss-Prot sequence database or the indicated references. The primary structures of the corresponding regions identified as lipid binding helix were collected. The included regions were checked for the extent of helicity either using the available crystallographic data and/or via secondary structure prediction using the program SOPMA [71], available at http://npsa-pbil.ibcp.fr/. In the 18-residue window at least 50% helicity of the sequence must be predicted.

### 3.2. Determination Lipid-Binding Potential

The lipid binding potential is performed as described before [12]. In essence, the mean hydrophobicity (<*H*>), the hydrophobic moment (μ*H*) and the net charge (*z*) were calculated. In the analysis, 18-residue windows were used, and for each sequence under investigation the window with the highest discrimination factor was selected. The ultimate classification rule renders the discrimination factor (*D*):

D=0.944 (<μH>)+0.33 (z)

When this discrimination factor is above 0.68, the corresponding can be considered to be a (potential) lipid-binding region. See [12] for detailed information about the way the discrimination factor is defined.

### 3.3. Eisenberg Plot Approach

The Eisenberg plot approach was essentially performed as described in the original study [1]. Both the mean hydrophobicity (<*H*>) and the hydrophobic moment (μ*H*) were extracted from the Heliquest program [6] and subsequently plotted. In the analysis, 18-residue windows were used. The basic difference with the original approach is the hydrophobicity scale used, which was the Fauchere and Pliska scale [16] instead of the original normalized ‘consensus’ scale by Eisenberg [2]. This study used the data set compiled by Eisenberg and co-workers [1,2]. The used segments are summarized in Table S1 and Table S2. The criteria used to select more recent examples were the presence of experimental evidence for the existence of protein–lipid or peptide–lipid interactions and the described use of the original Eisenberg plot methodology. The used segments are summarized in Table 1 and Table 2.

## 4. Conclusions

The data presented here indicates that Heliquest generated data can be utilized for a hydrophobic moment plot analysis. A comparison of both the original databases [1,2] used by Eisenberg and co-workers and the newly generated database (this study) of recent examples of well described lipid-binding proteins and peptides clearly demonstrates the validation of the Heliquest generated Eisenberg plot. One important advantage of the use Heliquest generated data plot is the fact that it utilizes a freely available and user-friendly software package [6].

During the introduction of the Eisenberg plot [1,2] there was consensus about the alpha-helical classification, either surface active, globular or transmembrane. The finding that numerous lipid-binding regions of experimentally demonstrated lipid-binding peptides and proteins were found to be located in the globular protein area of the Eisenberg plot is intriguing. The extension of the classical threefold classification has been postulated for the so-called oblique orientated α-helices [5,30,39]. For peptides, additional novel classes have been proposed such as the signal peptides [72], the helical antimicrobial peptides α-AMP [39,73] and cell-penetrating peptides [74]. For proteins, the new class is the amphitropic protein family [75–77]. Protein translocation motor proteins like SecA [12,78], BiP, and mtHsp70 [12] have been postulated to be members of this family. It seems that membrane dynamic processes involving proteins such as FtsY [12,50], Ffh [68] and Fis1 [67], are members of the amphitropic family. Taking all results together, it seems that protein classification has been significantly broadened since the introduction of the Eisenberg plot methodology.

There is a growing perception that membrane proteins can also possess the so-called non-annular lipid-binding sites, where specific anionic phospholipids bind tightly to the protein and have been demonstrated to be involved in the formation of homo-oligomeric structures [79] and hetero-oligomeric structures [80] of proteins. How these particular lipid-binding sites fit into the possible search for lipid-binding regions in proteins will be investigated in future investigations.

Based on all the sequences investigated in this study, a positive discrimination value above 80% was found for the Heliquest lipid binding discrimination factor, while the combined approach was able to identify all sequences as potential lipid binding. All sequences investigated were well reported examples of experimentally confirmed lipid-binding proteins or peptides. What the positive prediction value will be for not yet experimentally confirmed protein-lipid interacting proteins remains to be seen. This study clearly indicates however that the combined use of the Heliquest lipid binding discrimination factor and the Heliquest generated Eisenberg plot methodology provides a powerful tool for the search of possible lipid-binding regions in proteins. The presented bioinformatics approach might serve as a starting point for studying proteins which have not yet been characterized in detail when it comes to protein–lipid interactions.

## Supplementary Information



## Figures and Tables

**Table 1:** Representative examples of clearly identified transmembrane (M) and surface seeking (S) regions of proteins and peptides as reported in the literature in the period 1990–2010.

Name	Sequence	*z*	<*H*>	<μ*H*>	D	Conf.
RW16	RRWRRWWRRWWRRWRR	10	0.213	0.975	YES	S [17]
RL16	RRLRRLLRRLLRRLRR	10	0.006	0.824	YES	S [17]
Pbuy	FRKLFRVYSNFLRGKLKL	6	0.280	0.650	YES	S [18]
Pill	KQLIRFLKRLDRNLWGLA	4	0.447	0.633	YES	S [18]
Pc9k	NRLARHFRDIAGRVNQRL	4	0.096	0.591	YES	S [18]
Pqc7	LKDVEEAQQKIINIIRRL	1	0.280	0.650	YES	S [18]
Pc3c	WYSEMKRNVQRLERAIEE	0	0.113	0.615	NO	S [18]
Pihf	RDAKELVELFFEEIRRAL	−1	0.276	0.566	NO	S [18]
KL	KLLKLLLKLLKLLLKLLL	5	0.953	0.659	YES	S [19]
CRAMP18	GEKLKKIGQKIKNFFQKL	5	0.164	0.674	YES	S [19]
SPLN14–27	SLSRYAKLANRLA	3	0.254	0.530	YES	S [20]
SPLN28–41	PKLLETFLSKWIG	1	0.712	0.596	YES	S [20]
Histatin 5	SHAKRHHGYKRKFHEKHH	5	−0.157	0.263	YES	G [21]
PGLa^a^	SKAGAIA**G**KIAKVALKAL	3	0.398	0.501	YES	S [21]
SP-B(7–24)	YCWLCRALIKRIQAMIPK	4	0.747	0.434	YES	S [22]
PC-TP196-	VPNFLKDMARACQNYLKK	3	0.295	0.677	YES	S [23]
Equinatoxin II	ASLSFDILKTVLEALGNV	−1	0.591	0.458	NO	S [24]
KL4	KLLLLKLLLLKLLLLKLL	4	1.102	0.157	YES	M [25]
KALP23	KKLALALALALALALALA	2	0.783	0.154	YES	M [26]
WALP23	WWLALALALALALALALA	0	1.143	0.107	NO	M [26]
Glycophorin A (92–114)	ITLIIFGVMAGVIGTILLI	0	1.133	0.213	NO	M [27]
TMX31	WISFAISCFLLCVVLLGF	0	1.321	0.216	NO	M [28]
MHCClassII	VLVALLLAGQATTAYFLY	0	0.899	0.115	NO	M [29]

a This region is analyzed with a window of 11 AA in accordance to the original reference [21].

**Table 2:** Examples of demonstrated lipid-binding proteins and peptides, which are not always identified by the Eisenberg plot methodology. The results of using the lipid-binding discrimination factor of the Heliquest program are included.

Name	Sequence	*z*	<*H*>	<μ*H*>	D	Conf.
*SP & LBP:*						
1. prePhoE	KKSTLALVVMGIVASASV	2	0.558	0.045	Y	[31]
2. preLamB	RKLPLAVAVAAGVMSAQA	2	0.478	0.157	Y	[32]
3. proOmpA	KKTAIAIAVALAGFATVA	2	0.569	0.204	Y	[33]
4. prePhoA	TIALALLPLLPTPVTKAR	2	0.744	0.197	Y	[34]
5. Ovalbumin	IFYCPIAIMSALAMVTLG	0	1.036	0.165	N	[35]
6. Aurein 1.2	GLFDIKKVASVIGGL	1	0.583	0.326	N	[36,37]
7. Citropin	GLFDVIKKVASVIGGL	1	0.623	0.614	Y	[36,37]
8. Maculatin 1.1	GLFGVLAKVAAHVVPAIA	1	0.738	0.408	Y	[36,37]
9. VP1	GTAMRILGGVI	1	0.665	0.468	Y	[38]
10. HA2 FP	FGAIAGFIENGWEGMIDG	−3	0.579	0.533	N	[38]
11. AP1	GEQGALAQFGEWL	−2	0.488	0.399	N	[39]
12. SIV peptide	GVFVLGFLGFLA	0	1.102	0.259	N	[40]
13. Gaegurin 5	LGALFKVASKVLPSVCAI	2	0.749	0.463	Y	[41]
14. PBP5	GNFFGKIIDYIKLMFHHW	1	0.768	0.616	Y	[42]
16. Penetratin	RQIKIWFQNRRMKWKK	7	0.193	0.327	Y	[43]
17. Polyarginine-R9	RRRRRRRRR	9	−1.010	0.146	Y	[44]
18. Substance-P	RPKPQQFFGLM	2	0.501	0.298	Y	[45]
19. Dermaseptin B2	**I**KE**V**GKEAAKAAAKAAGK	3	−0.058	0.395	Y	[46]
*Amphitropics:*						
20. SecA(1–21)	MLIKLLTKVFGSRNDRTL	3	0.442	0.303	Y	[14]
21. SecA(108–125)	KTLTATLPAYLNALTGKG	2	0.437	0.352	Y	[47]
22. SecA(593–614)	ALMRIFASDRVSGMMRKL	3	0.425	0.131	Y	[48]
23. SecA(865–882)	AAAAALAAQTGERKVGRN	2	0.049	0.088	Y	[14]
24. FtsY(1–18)	MAKEKKRGFFSWLGFGQK	4	0.277	0.332	Y	[49]
25. FtsY(188–208)	KPTKEGFFARLKRSLLKT	5	0.198	0.254	Y	[50]
26. Apocyt c2–21	VEKGKKIFVQKCAQCHTV	3	0.333	0.341	Y	[51]
27. Apocyt c80–101	AGIKKKTEREDLIAYLKK	3	0.046	0.129	Y	[51]
28. EcMinD251–269	RPFRFIEEEKKGFLKRLF	3	0.287	0.498	Y	[52]
29. α-synuclein1–15	MDVFMKGLSKAKEGV	1	0.285	0.517	Y	[53]
30. ARF1	MGNIFANLFKGLFGKKEM	2	0.474	0.400	Y	[54]
31. K-segment dehydrins	EKKGIMDKIKEKLPG	2	0.017	0.363	Y	[55]
*Miscellaneous:*						
32. Kes 1p (7–29)	SSSWTSFLKSIASFNGDL	0	0.500	0.523	N	[56]
33. PBP4	RRIPLVRFESRLYKDIYQNN	3	0.331	0.285	Y	[42]
34. KCNQ1354–372	KVQQKQRQKHFNRQIPAA	5	−0.023	0.154	Y	[57]
35. ABP280(49–71)	FTRWCNEHLKCVSKRIAN	3	0.370	0.560	Y	[58]
36. L15K7	KLLKLLLKLLKLLLKLLLKLLK	5	0.953	0.659	Y	[59]

**Table 3:** Examples of the use of a combined Heliquest discrimination factor and a Heliquest generated Eisenberg plot methodology in the identification of potential lipid-binding regions.

Name	Sequence	*z*	<*H*>	<μ*H*>	D	Confirmed
M13 coat protein:						
	_2_KKSLVLKASVAVATLVPM_19_	3	0.559	0.072	YES	[65]
	_47_YAWAMVVVIVGATIGIKL_64_	1	0.923	0.062	NO	[65]
	_54_VIVGATIGIKLFKKFTSK_71_	4	0.553	0.288	YES	-
Ffh:						
(P0AGD7)	_1_MFDNLTDRLSRTLRNISG_18_	1	0.255	0.663	YES	-
	_44_ALPVVREFINRVKEKAVG_61_	2	0.313	0.365	YES	-
	_166_QKPVDIVNAALKEAKLKF_183_	2	0.272	0.331	YES	-
	_309_SKVDRAQAEKLASKLKKG_326_	4	−0.118	0.297	YES	-
	_336_EQLRQMKNMGGMASLMGK_353_	2	0.218	0.261	YES	-
	_395_KGSRKRRIAAGCGMQVQD_412_	4	0.008	0.140	YES	-
	_415_RLLKQFDDMQRMMKKMKK_432_	5	0.064	0.606	YES	-
	_428_KKMKKGGMAKMMRSMKGM_445_	7	0.039	0.327	YES	-
Fis1:						
(P40515)	_35_PTATIQSRFNYAWGLIKS_52_	2	0.514	0.349	YES	[67]
	_60_LGVKILTDIYKEAESRRR_77_	2	0.147	0.326	YES	-
	_108_RNNKQVGALKSMVEDKIQ_125_	2	0.023	0.305	YES	-
	_133_VVAGGVLAGAVAVASFFL_150_	0	0.811	0.145	YES	[67]
