# Supplementary material for: New User-Friendly Approach to Obtain an Eisenberg Plot and Its Use as a Practical Tool in Protein Sequence Analysis
Source: Int J Mol Sci. 2011 Aug 30;12(9):5577–91. doi: 10.3390/ijms12095577 (PMC3189734; doi:10.3390/ijms12095577)
Supplement: Supplementary file 1 [file ijms-12-05577-s001.pdf]

Supplementary Information

Rob C.A. Keller

Section Chemistry, Charlemagne College, Wilhelminastraat 13-15, 6524 AJ Nijmegen, The Netherlands; E-Mail: rcakeller@kpnmail.nl; Tel.: +0031-243820460; Fax: +0031243820460

Received: 25 July 2011; in revised form: 22 August 2011 / Accepted: 22 August 2011 / Published: 30 August 2011

Abstract: The Eisenberg plot or hydrophobic moment plot methodology is one of the most frequently used methods of bioinformatics. Bioinformatics is more and more recognised as a helpful tool in Life Sciences in general and recent developments in approaches recognizing lipid binding regions in proteins are promising in this respect. In this study a bioinformatics approach specialized in identifying lipid binding helical regions in proteins was used to obtain an Eisenberg plot. The validity of the Heliquest generated hydrophobic moment plot were checked and exemplified. This study indicates that the Eisenberg plot methodology can be transferred to another hydrophobicity scale and renders a user-friendly approach which can be utilized in routine checks in protein-lipid interaction and in protein and peptide lipid binding characterization studies. A combined approach seems to be advantageous and results in a powerful tool in the search of helical lipid-binding regions in protein and peptides. The strength and limitations of the Eisenberg plot approach itself are discussed as well. The presented approach not only leads to a better understanding of the nature of the protein-lipid interactions and provides a user-friendly tool for the search of lipid-binding regions in proteins and peptides.

Keywords: Amphitropic proteins; Eisenberg plot; Hydrophobic moment plot; Heliquest; Lipid Binding Regions; Protein-lipid interactions; Transmembrane proteins .

Table S1. Helices included in the Heliquest generated hydrophobic moment plot. Overview of results extracted from the database constructed by Eisenberg *et al.* (1982).

Protein	Sequence	1st residue	<*H*>	<μ*H*>
Globular:				
Adenylate Kinase	P**L**ETVLDMLRDAMVAKVDTS	69(Leu)	0.412	0.375
(P00571)	P**E**TMTKRLLKRGETSGRVDD	123(Glu)	-0.026	0.275
	F**E**KINEGFDLLRSGESIRTIL	353(Glu)	0.214	0.324
	S**V**DDVFSQVCTHLDTLK	179(Val)	0.467	0.506
Alcohol dehydrogenase	**C**LIGCGFSTGYGSAVKVAK	170(Cys)	0.628	0.129
(P00327-1)	K**S**KDSVPKLVADFMAKKFAL	202(Gly)	0.557	0.079
	K**S**KDSVPKLVADFMAKKFAL	324(Ser)	0.182	0.303
	F**E**KINEGFDLLRSGESIRTIL	353(Glu)	0.214	0.324
Carboxypeptidase A	H**T**LDEIYDFMDLLVAEHPQLV	14(Thr)	0.539	0.415
(P00730)	T**N**PDGFAFTHSQNRLWRKTR	112(Asn)	0.221	0.236
	S**E**VEVKSIVDFVKDHGNFKA	173(Glu)	0.220	0.336
	P**D**KTELNQVAKSAVEALKSL	215(Asp)	0.062	0.324
	S**Q**IIPTAQETWLGVLTIMEH	285(Gln)	0.755	0.365
Chymotrypsin<br>(P00766)	A**R**VTALVNWVQQTLAAN	230(Arg)	0.507	0.290
HA2 haemagglutinin<br>(P03437)	QDLL**E**KYVEDTKIDLWSYNAE	77(Glu)	0.282	0.239
Lactate dehydrogonase	A**V**GMACAISILMKDLADEVA	32(Val)	0.569	0.332
(P00344)	V**M**EDKLKGEMMDLQHGSLF	55(Met)	0.314	0.151
	QQEG**E**SRLNLVQRNVNIFKF	107(Glu)	0.541	0.282
	K**F**IIPDIVKHSPDCIILVVS	120(Phe)	0.927	0.259
	SG**C**NLDSARFRYLMGERLGVHS	165(Cys)	0.393	0.137
	TS**W**AIGLSVADLAETIMKNLCR	249(Trp)	0.593	0.259
	KL**K**PDEEQQLQKSATTLWDIQK	308(Lys)	0.177	0.260
Myoglobin	M**V**LSEGEWQLVLHVWAKVEAD	1(Val)	0.653	0.242
(P02185)	A**D**VAGHGQDILIRLFKSHPETL	20(Asp)	0.417	0.401
	A**S**EDLKKHGVTVLTALGAIL	58(Ser)	0.399	0.362
	I**P**IKYLEFISEAIIHVLHSRHP	100(Pro)	0.751	0.434
	G**A**DAQGAMNKALELFRKDI	125(Ala)	0.193	0.448
Myohaem Erythrin	F**Y**QLDEEHKKIFKGIFDCI	18(Tyr)	0.446	0.442
(P02247-1)	N**S**APNLATLVKVTTNHFTH	40(Ser)	0.310	0.435
	S**E**VVPHKKMHKDFLEKIGG	69(Glu)	0.161	0.336
	D**A**KNVDYCKEWLVNHIKGT	93(Ala)	0.423	0.327
Ribonuclease S	E**T**AAAKFERQHMDSSTSAASSS	3(Thr)	0.082	0.189
(P61823-1)	S**N**YCNQMMKSRNLTKDRCKPV	24(Asn)	0.036	0.330
Thermolysin	YD**A**PAVDAHYYAGVTYDYYKN	67(Asp)	0.404	0.123
(P00800)	G**I**DVVAHELTHAVTDYTAGLIY	137(Ile)	0.439	0.318
	IY**Q**NES**G**AINEAISDIFGTLVE	160(Glu)	0.424	0.473
	S**G**IINKAAYLISQGGTHYGVS	235(Gly)	0.454	0.307
	G**R**DKLGKIFYRALTQYLTPTSN	260(Arg)	0.398	0.396
	N**F**SQLRAAAVQSATDLYGSTS	281(Phe)	0.363	0.276
	TS**Q**EVASVKQAFDAVGVK	301(Gln)	0.219	0.218

Table S1. *Cont.*

Protein	Sequence	1st residue	<*H*>	<μ*H*>
TMV coat protein (P03571)	AD**P**IELINLCTNALGNQFQT	20(Pro)	0.594	0.450
	QT**Q**QARTVVQRQFSEVWKPS	38(Gln)	0.243	0.336
	YN**A**VLDPLVTALLGAFDTRN	74(Ala)	0.558	0.294
	RR**V**DDATVAIRSAINNLIVE	114(Val)	0.418	0.365
Triose phosphate Isomerise (P00940)	GD**K**KSLGELIHTLNGAKLSAD	17(Lys)	0.236	0.230
	A**P**SIYLDFARQKLDAKIGVAA	44(Pro)	0.418	0.166
	E**S**DELIGQKVAHALAEGLGVI	105(Ser)	0.327	0.180
	G**I**TEKVVFEQTKAIADNVKDW	138(Ile)	0.242	0.327
	A**T**PQQAQEVHEKLRGWLKSH	177(Thr)	0.184	0.466
	V**T**GGNCKELASQHDVDGFLVG	213(Thr)	0.257	0.059
Membrane:				
Glycophorin (P02724)	**I**TLIIFGVMAGVIGTILLISYG	12(Ile)	1.133	0.213
Glycoprotein (P03522)	**I**ASFFFIIGLIIGLFLVGIH	51(Ser)	1.264	0.245
Hemagglutinin (P03437)	**W**ISFAISCFLLCVVLLGFI	185(Trp)	1.321	0.216
Hemagglutinin (P03451)	**V**YQILAIYATVAGSLSLAIM	527(Val)	0.792	0.234
IgM (P01871)	**N**LWATASTFIVLFLLSLFY	569(Asn)	1.089	0.285
Isomaltase (P07768)	**I**TLIVLFVIVFIIAIALIAV	10(Ile)	1.434	0.119
M13 coat (P69541)	AMVVVIVGATIGIKLFKKF**T**	20(Tyr)	0.746	0.207
M13 procoat (P69541)	**S**LVLKASVAVATLVPMLS	-20(Ser)	0.762	0.204
Surface active:				
δ-Haemolysin (Q512D3)	**M**AGDIISTIVDFIKLIAETV	1(Met)	0.701	0.578
δ-Haemolysin (P0C1V1)	**M**AQDIISTIGDLVKWIIDTV	1(Met)	0.689	0.583
Melittin (P01501)	**G**IGAVLKVLTTGLPALISWIK	1(Gly)	0.759	0.456
Melittin (P01504)	**G**IGAILKVLATGLPTLISWIK	1(Gly)	0.792	0.477
Cytotoxic peptide (see ref.)	**L**LQSLLSLLQSLLSLLLQWLK	1(Leu)	0.993	0.545
Diphtheria toxin (fragment B) (P00578)	**L**VGELVDIGFAAYNFVES	7(Leu)	0.629	0.360

Table S2. Helices included in the Heliquest generated hydrophobic moment plot. Overview of results extracted from the database constructed by Eisenberg *et al*. (1984).

Protein	Sequence	1st residue	<*H*>	<μ*H*>
A. *Surface region:*				
Cecropin A<br>(P01507)	**K**LFKKIEKVGQNIRDGII	3(Lys)	0.262	0.619
Cecropin B<br>(P01508)	**K**VFKKIEKMGRNIRNGIV	3(Lys)	0.169	0.635
δ-Haemolysin<br>(Q512D3)	TIVDFIKL**I**AETVKKFTK	16(Ile)	0.491	0.671
δ-Haemolysin<br>(P0C1V1)	TIGDLVKW**I**IDTVNKFTKK	16(Ile)	0.514	0.654
Melittin<br>(P01501)	VLKVLTT**G**LPALISWIKR	12(Gly)	0.756	0.545
Melittin<br>(P01504)	ILKVLAT**G**LPTLISWIKN	12(Gly)	0.811	0.554
Cytotoxic peptide<br>(see ref.)	LLSLLQS**L**LSLLLQWLKR	12(Leu)	0.927	0.620
Cytotoxic peptide<br>(see ref.)	**L**QSLLSLLQSLLSLLLQW	2(Leu)	1.024	0.562
B. *Transmembrane region:*				
Mouse class I H-2d no.1<br>(P01899)	IVAVLGVLGA**M**IIGAVVA	C-43(Met)	0.965	0.264
H-2k<br>(P04223)	VIIAV**L**VVLGAAIVTGAVV	C-59(Leu)	0.979	0.167
Human class I HLA<br>(P01892)	VG**I**IAGLVLFGAVITGAVV	C-55(Ile)	0.926	0.238
Mouse class II A_{α}<br>(P01910)	TVVCALGLS**V**GLVGIVVGTIFI	C-29(Val)	0.905	0.124
A_{β}<br>(P01921)	LSGIGGCVLGVIGFLGLGLFI	C-78(Leu)	0.896	0.293
E_{α}<br>(P01904)	N**V**MCALGLFVGLVGIVVGII	C-38(Val)	0.959	0.216
IgD (delta)<br>(P01882)	GLWPTMCTFVALFL**L**TLL	C-15(Leu)	1.213	0.291
IgG1 (gamma 1)<br>(P01869)	GLWITITI**F**ISLFLLSVC	C-45(Phe)	1.279	0.188
IgM (mu)<br>(P01871-2)	F**I**VLFLLSLFYSTTVTLF	C-20(Ile)	1.198	0.122
Human class II DC1<br>(P01909)	LGLS**V**GLVGIVVGTVFII	C-29(Val)	1.034	0.169
DRα<br>(P01903)	LGLT**V**GLVGIIIGTIFII	C-29(Val)	1.147	0.242
DRβ	GVGGFV**L**GLLFLGAGLFI	C-30(Leu)	1.023	0.151

Table S2. *Cont.*

Protein	Sequence	1st residue	<*H*>	<μ*H*>
(P01911)				
Acetylcholine receptor	PLYFVVNVI**I**PCLLFSFL	244(Ile)	1.263	0.081
α-subunit:				
(P02710)	MTLSISVLLSL**T**VFLLVI	278(Thr)	1.160	0.131
	L**F**TMIFVISSIIITVVVI	304(Phe)	1.257	0.216
	ILLC**V**FMLICIIGTVSVF	437(Val)	1.337	0.116
β-subunit:	PL**F**YIVYTIIPCILISIL	243(Phe)	1.335	0.193
(P02712)	MSLSISALL**A**VTVFLLLL	282(Ala)	1.107	0.073
	LMFIM**I**LVAFSVILSVVVL	313(Ile)	1.271	0.171
	LFLY**V**FFVICSIGTFSIF	466(Val)	1.271	0.022
γ-subunit:	**L**FYIINIIAPCVLISSLV	237(Leu)	1.177	0.151
(P02714)	CT**L**SISVLLAQTIFLFLI	271(Leu)	1.154	0.055
	I**F**VMFVSMLIVMNCVIVL	306(Phe)	1.282	0.115
	CFW**I**ALLLFSIGTLAIFL	471(Ile)	1.328	0.150
δ-subunit:	P**L**FYVINFITPCVLISFL	247(Leu)	1.215	0.077
(P02718)	MSTAISVLLAQAVFLLLT	276(Glu)	0.939	0.160
	**L**MFIMSLVTGVIVNCGIV	313(Leu)	1.061	0.221
	MF**I**ITPVMVLGTIFIFVM	481(Ile)	1.270	0.127
Bacteriorhodopsin:	IWLA**L**GTALMGLGTLYFLV	15(Leu)	1.076	0.186
(P02945)	**F**YAITTLVPAIAFTMYLSMLL	42(Phe)	1.051	0.055
	YWARYADWLFTTPL**L**LLD	93(Leu)	0.890	0.360
	ILALVG**A**DGIMIGTGLVGAL	114(Ala)	0.793	0.147
	**V**WWAISTAAMLYILYVLFF	136(Val)	1.207	0.133
	LRNVTVVLW**S**AYPVVWLI	183(Ser)	1.006	0.199
	**I**ETLLFMVLDVSAKVGFGLIL	203(Ile)	0.960	0.153
Rhodopsin:	MFLLI**M**LGFPINFLTLYV	49(Met)	1.249	0.182
(P02699)	YILLNLA**V**ADLFMVFGGF	81(Val)	0.992	0.218
	**G**FFATLGGEIALWSLVVLAI	114(Gly)	0.966	0.239
	IMGVAFTW**V**MALACAAPP	162(Val)	0.957	0.118
	IYMFV**V**HFIIPLIVIFFC	210(Val)	1.450	0.135
	MVIIM**V**IAFLICWLPYAG	258(Val)	1.299	0.104
	**I**FMTIPAFFAKTSAVYNPVIYI	286(Ile)	0.797	0.305

© 2011 by the authors; licensee MDPI, Basel, Switzerland. This article is an open access article distributed under the terms and conditions of the Creative Commons Attribution license (http://creativecommons.org/licenses/by/3.0/).
